# Zika virus differentially infects human neural progenitor cells according to their state of differentiation and dysregulates neurogenesis through the Notch pathway

**DOI:** 10.1080/22221751.2019.1637283

**Published:** 2019-07-08

**Authors:** Pauline Ferraris, Marielle Cochet, Rodolphe Hamel, Ivan Gladwyn-Ng, Christian Alfano, Fodé Diop, Déborah Garcia, Loïc Talignani, Claudia N. Montero-Menei, Antoine Nougairède, Hans Yssel, Laurent Nguyen, Muriel Coulpier, Dorothée Missé

**Affiliations:** a MIVEGEC, IRD, Univ. Montpellier, CNRS, Montpellier, France; b UMR1161 Virologie, ANSES, INRA, Ecole Nationale Vétérinaire d'Alfort, Université Paris-Est, Maisons-Alfort, France; c GIGA-Neuroscience, Interdisciplinary Cluster for Applied Genoproteomics (GIGA-R), University of Liège, C.H.U. Sart Tilman, Liège, Belgium; d CRCINA, INSERM, Université de Nantes, Université d’Angers, Angers, France; e UVE, Aix Marseille Univ-IRD 190, Inserm 1207-IHU Méditerranée Infection, Marseille, France; f Centre d’Immunologie et des Maladies Infectieuses, Inserm, U1135, Sorbonne Universités, UPMC, APHP Hôpital Pitié-Salpêtrière, Paris, France

**Keywords:** Zika, arbovirus, human neural progenitor, Notch, neurogenesis, flavivirus

## Abstract

Zika virus (ZIKV) is a mosquito-borne Flavivirus that causes Zika disease with particular neurological complications, including Guillain-Barré Syndrome and congenital microcephaly. Although ZIKV has been shown to directly infect human neural progenitor cells (hNPCs), thereby decreasing their viability and growth, it is as yet unknown which of the cellular pathways involved in the disruption of neurogenesis are affected following ZIKV infection. By comparing the effect of two ZIKV strains *in vitro* on hNPCs, the differentiation process of the latter cells was found to lead to a decreased susceptibility to infection and cell death induced by each of the ZIKV strains, which was associated with an earlier and stronger antiviral innate immune response in infected, differentiated hNPCs, as compared to undifferentiated cells. Moreover, ZIKV modulated, both in hNPCs and *in vivo* in fetal brain in an experimental mouse model, the expression of the Notch pathway which is involved in cellular proliferation, apoptosis and differentiation during neurogenesis. These results show that the differentiation state of hNPCs is a significant factor contributing to the outcome of ZIKV infection and furthermore suggest that ZIKV infection might initiate early activation of the Notch pathway resulting in an abnormal differentiation process, implicated in ZIKV-induced brain injury.

## Introduction

ZIKA virus (ZIKV) is a newly emerging arbovirus, which belongs to the *Flaviviridae* family. Since its discovery in Africa, the virus has spread throughout the Pacific and Latin America, emphasizing the capacity of ZIKV to spread to non-endemic regions worldwide [1]. There is a significant risk of viral spreading to yet unaffected EU Countries, Territories and Outermost Regions due to the presence of competent vectors and the movement of populations within and between these regions. Several imported cases from South America and the Caribbean have been reported in North America, Australia and Europe [2]. About 80% of people affected by ZIKV are asymptomatic and the pathology is generally mild. However, during the French Polynesian outbreak, an increased incidence of Guillain-Barré Syndrome (GBS) was reported [3]. This unusual increase in GBS, concomitant to ZIKV circulation, was also reported in several countries in Latin America [4,5]. Of particular concern are reports of microcephaly in newborns, a neurological complication that results in the failure of the brain to develop properly [6–8]. A sharp rise in the incidence of pregnancy-associated microcephaly linked to a concurrent epidemic of ZIKV infection occurred between 2014 and 2016 [9] and epidemiological evidence suggests that congenital abnormalities and fetal demise in pregnant women as a result of ZIKV infection is steadily on the rise [10–13]. Studies on the French Polynesian and Latin American outbreaks revealed congenital abnormalities associated with ZIKV infection [7], suggesting that ZIKV strains in both regions have the potential to cause disease during pregnancy. In Sub-Saharan Africa and Asia however, there is no evidence of ZIKV-related complications, although the virus has been circulating for decades. In cases of microcephaly and spontaneous abortion associated with ZIKV infection, viral RNA and antigens were detected in the brains of infected fetuses and newborns [6,14], an observation that corroborates the results of several studies showing that ZIKV targets human brain cells [15–21], thereby reducing their viability and growth as neurospheres and brain organoids [16]. Together, these results suggest that ZIKV affects human brain development by abrogating neurogenesis. ZIKV has also been shown to directly infect human cortical neural progenitor cells, causing transcriptional dysregulation and attenuated cell growth, often with cytotoxic effects [10,17,21]. Nevertheless, the impact of ZIKV infection on hNPC in the process of cellular differentiation is not well-characterized.

Moreover, many questions remain unanswered regarding the neurological complications caused by different primary isolates of ZIKV. It has also been shown that ZIKV infection induces an innate immune response in different cell types through the activation of IFN signalling pathways [15,22–24]. The induction of an innate immune response can be associated with neuro-pathogenesis directly by inducing neuro-inflammation, as recently reported with the activation of TLR3 associated with NPC depletion in human organoids [20], and/or by ZIKV interaction with STAT2, thereby inhibiting IFN activity [24,25]. In this study, we have investigated the nature of the cellular signalling pathways involved in ZIKV neuropathogenicity, following the infection of human fetal brain-derived primary neural progenitor cells (hNPCs) at different states of differentiation. We show that ZIKV tropism is affected by the state of differentiation of hNPCs and that ZIKV infection modulates the Notch pathway during neurogenesis.

## Materials and methods

### Ethics statement

Human fetuses were obtained after a legal abortion with the written informed consent of the patient. The procedure for the procurement and use of human fetal CNS tissue was approved and monitored by the “Comité Consultatif de Protection des Personnes dans la Recherche Biomédicale” of Henri Mondor Hospital, France. The cells are declared at the “Centre des Ressources Biologiques” of the University Hospital in Angers with reference numbers at the Research Ministry: declaration No DC-2011-1467; authorization No AC-2012-1507.

All animals used in this study were housed under standard conditions within a specific-pathogen-free facility at GIGA, C.H.U, Sart Tilman, Belgium, with *ad libitum* access to food and water. All animal procedures were approved by the Animal Ethics Committee of the Liege University (#16-1829) and are compliant with the Belgian Ministry of Agriculture, in agreement with the European Community Laboratory Animal Care and Use Regulations (86/609/CEE, Journal Officiel des Communautés Européennes L358, 18 December 1986). Time-mated immunocompetent adult SWISS mice (2–3 months of age, purchased from Janvier Laboratories, France), were allowed to acclimate for at least 24 h (hrs) prior to any procedure and were used when noon of the day after mating was considered embryonic day 0.5 (E0.5).

### Viral isolates

The low-passage-number PF-25013-18 Asian strain of ZIKV (ZIKV**^As^** strain, obtained via V. M. Cao-Lormeau and D. Musso, Institut Louis Malardé [ILM], Tahiti Island, French Polynesia), isolated from a viremic patient in French Polynesia in 2013 (PF13) [26], and an African strain HD787888 (ZIKV^Af^ strain, obtained via A.A Sall, Pasteur Institute, Dakar) isolated from a viremic patient in Senegal in 1991 [27] were used in the current study and were grown in *A. albopictus* C6/36 mosquito cells as described previously [22].

### Human neural progenitor cells

HNPCs were prepared from the central nervous system of first-trimester human fetuses, as described previously [28]. Medium, referred to as N2A medium, was changed 3 times a week, and the growth factors EGF and bFGF (both at 20 ng/ml; Abcys, Eurobio, France) were added to maintain undifferentiated cells. The population of undifferentiated cells is composed exclusively by hNPCs. The D0 cell populations are composed mainly by hNPCs and in a smaller proportion of neurons and astrocytes whereas the D5 cells are mainly composed of neurons and astrocytes.

### ZIKV infection of cells

For infection, hNPCs were seeded in culture plates at 4 × 10^4 ^cells per cm^2^. The cells were then rinsed with phosphate-buffered saline (PBS), and ZIKV was diluted to the desired multiplicity of infection (MOI) of 0.5 and added to the cells. The cells were incubated for 2 h at 37°C with gentle agitation. The inoculum was removed and the cells were washed twice with PBS. Culture medium with or without Notch signalling inhibitor DAPT (final concentration of 75 µM), was added to each well, and cells were incubated at 37°C and 5% CO_2_ for the duration of the experiment. As a control, hNPCs were incubated with the culture supernatant from uninfected C6/36 cells, referred to as mock-infected cells. Cells and supernatants were collected from 24 hpi to 96 hpi to get an overview of the infection.

### Animal surgery

All procedures with ZIKV were conducted within a BSL2. Preoperative analgesia was administered subcutaneously with Temgesic (buprenorphine 0.1 mg/kg body weight, Merck, Brussels, Belgium) before induction of anaesthesia with isoflurane (Abbot Laboratories Ltd, Kent, UK) in a transparent oxygen carrier. Following the testing for absent pain and deep reflexes, a mini-laparotomy was performed with a 1.0- to 1.5-cm incision through the lower ventral peritoneum; the uterine horns were careful extracted onto warm humidified gauze pads. Each E12.5 embryo was visualized with a cold-halogen lamp and randomly assigned to receive intra-cerebro-ventricular (ICV) injection 0.5–1.0 µl of either mock media or ZIKV (strain H/PF/2013, 1.6 × 107 TCID 50/mL), according to previous publications [29]. The female mice were euthanised by neck dislocation on the day of harvesting for embryos at E14.5

### Animal monitoring and euthanasia

The time-mated murine dams were monitored daily from the morning on their respective surgery until the day of euthanasia. Their health status was assessed based on two scores that were adapted from Burkholder and co-workers [29,30], and tabulated in ordinal scales of their body condition, as well as pain and distress. In addition, all mice were weighed daily.

### ZIKV plaque assay

To quantify viral production the supernatant of infected cells was collected from 24 hpi to 96 hpi at, to get an overview of the infection. Cells were then infected as described previously [31]. This experiment was repeated three times.

### Proliferation test

To quantify hNPC viability and proliferation, ZIKV and mock-infected undifferentiated, D0- and D5-hNPCs were plated at a density of 15000 cells/well in 96-well plates (Falcon, Corning, France) and maintained in culture from 24 hpi to 96 hpi at 37°C, to get an overview of the infection. Wst1 Cell Proliferation Assay (Roche, France) was used to quantify the cell metabolic activity, at each time point, as described previously [28].

### Real-time PCR gene analysis

In order to performed transcriptomic analysis, infected and uninfected hNPCs were harvested at the early stages of the infection (24 and 48 hpi). Total RNA was purified from hNPCs using Nucleospin® RNA/protein kit (Macherey Nagel, Hoerdt, France) according to the manufacturer’s instructions. cDNA was synthesized using 1 μg of purified RNA and an M-MLV reverse transcription kit (Promega, Charbonière, France) with random hexamers, following the manufacturer’s protocol. Gene expression was quantified using real-time PCR with an Applied Biosystems 7300 real-time PCR system. Real-time PCR was performed as described previously [22]. Primers of genes targeted are listed in supplemental Table 1. GAPDH mRNA was used as an endogenous control.

### Western blotting

In order to performed transcriptomic analysis, infected and uninfected hNPCs were harvested at the early stages of the infection (24 and 48 hpi).Total protein was extracted from hNPCs using Nucleospin® RNA/protein kit (Macherey Nagel), following the manufacturer’s protocol. Total protein was obtained from microdissected embryonic mouse brains and neuronal progenitors seeded in 6-well culture dishes after application of Trizol, adapted according to previously published protocols [29]. Protein concentration was determined using a protein quantification assay kit (Macherey Nagel) following the manufacturer’s protocol. Equal amounts of proteins were mixed with Nupage sample buffer, subjected to SDS-PAGE, and electrotransferred onto a nitrocellulose membrane. The membrane was blocked with PBS-0.1% Tween 20 containing 5% Bovin Serum Albumin (BSA) and incubated overnight at 4°C with primary antibodies. The following antibodies were used: anti-Notch1, anti-activated Notch, anti-RBPSUH, anti-MAML1, anti-p21, anti-Hes1, anti-STAT1, anti-pSTAT1, anti-STAT2, anti-pSTAT2, was purchased from Cell signalling (Cell Signaling Technology, Inc., Danvers, MA, USA); anti-PKR (Santa Cruz,Dallas, Texas, USA); anti-MX1, anti-IFIT3 (GeneTex, Irvine, CA, USA); anti-NS5 (a kind gift from Dr. A. Merit) and anti-actin (Sigma, St. Louis, Missouri, USA). Membranes were washed three times with PBS-Tween, and subsequently incubated for 1 h at room temperature with horseradish peroxidase-coupled secondary antibodies in PBS-Tween containing 1% skim milk. Membranes were again washed three times, and proteins were detected with chemiluminescence using an ECL plus chemiluminescent substrate kit (Thermo Scientific, Courtaboeuf, France) and Typhoon 9500 (Healthcare Life Sciences, Velizy-Villacoublay, France). Protein expression levels were quantified using IMAGEJ software (National Institutes of Health, USA).

### Neurogenesis RT^2^ profiler PCR array

In order to performed transcriptomic analysis, infected and uninfected hNPCs were harvested at the early stages of the infection (24 and 48 hpi). Total RNA was extracted from hNPCs using Tri-reagent (Sigma, Saint Quentin Fallavier, France) according to the manufacturer’s instructions. The concentrations of all RNA samples were assessed using a NanoDrop spectrophotometer (NanoDrop Technologies, Wilmington, DE). The same amount of total RNA (400 ng) from each sample was subjected to a cDNA synthesis reaction using an RT2 First Strand kit (Qiagen, Valencia, CA), following the protocol described previously [22].

### Immunofluorescence

For immunofluorescence, cells were fixed, in 3.7% paraformaldehyde for 20 min at room temperature and washed in PBS, at 72 hpi to allow enough differentiation to be counted and before the cytotoxicity effect. Immunostaining were performed as described previously [22,28]. Images were acquired with an inverted EVOS®FL microscope Cell Imaging system (Life Technologies, Carlsbad, California, United States), then processed and analyzed in IMAGEJ (National Institutes of Health). To quantify the different cell populations and infected cells, astrocytes, neurons and total cells were counted within 10 independent fields of 0.2 mm^2^.

### Statistical analyses

All data (means ± standard errors of the means [SEM]) are based on a minimum of three independent experiments or four infected or uninfected animals for each result shown. Each statistical test is described in the figure legend. Data were analyzed using student T-tests, one-way ANOVAs (comparing to one control group) or Tuckey’s range test (comparing two groups) and Mann–Whitney test (comparing two groups) with a *P*-value significant when *p < 0.05, **p < 0.01, ***p < 0.001.

## Results

### Viral production in ZIKV-infected hNPCs undergoing neuronal differentiation and cytotoxic effects of ZIKV infection

**Figure 1.**
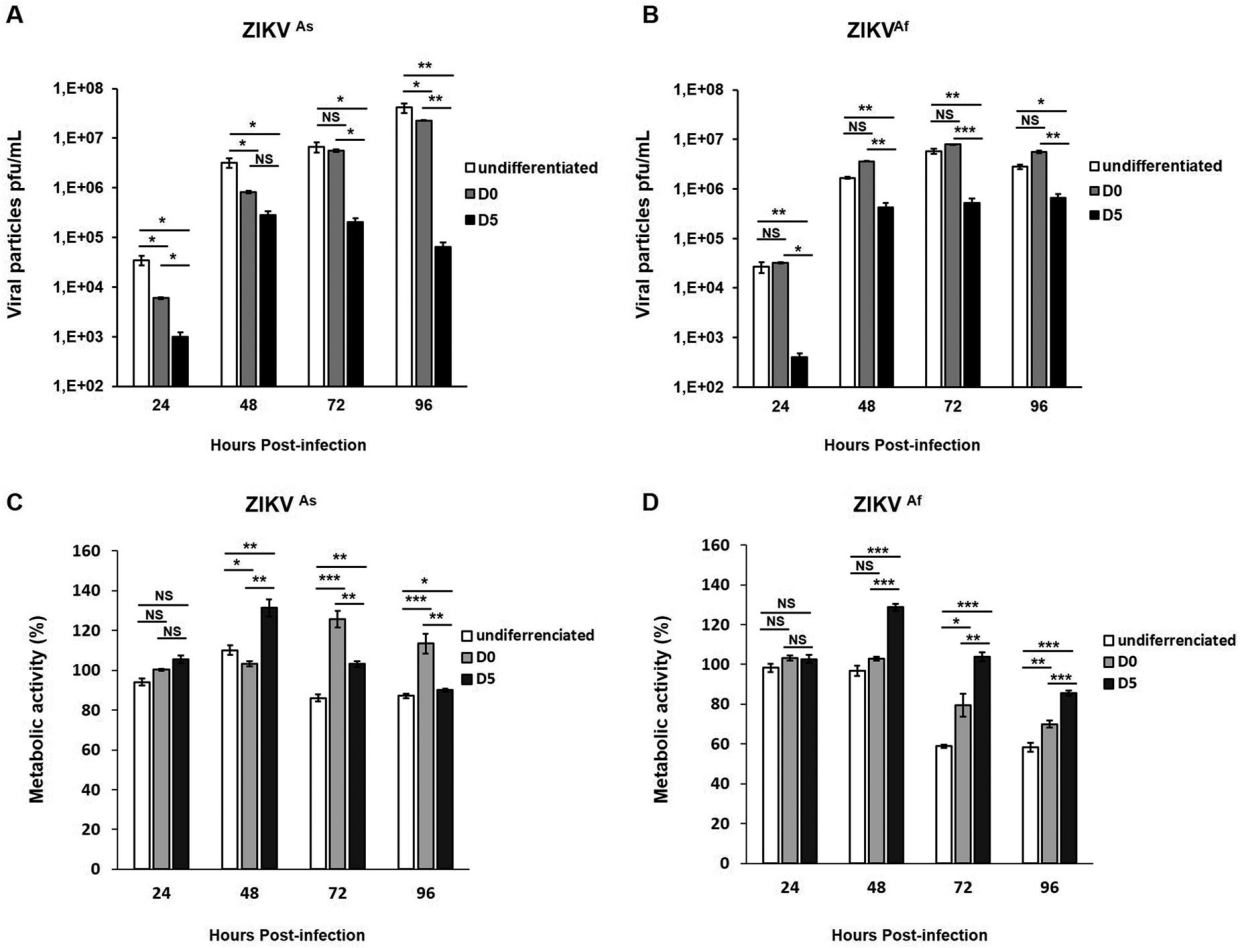
ZIKV-induced viral production and cytotoxic effects in undifferentiated and differentiated hNPCs. Undifferentiated, D0- and D5-hNPCs were infected with the ZIKV^As^ or ZIKV^Af^ strain, at MOI 0.5. Culture supernatants were collected from 24 hpi to 96 hpi to determine the viral titer and metabolic activity. Viral titers of the (**A**) ZIKV^As^ strain and (**B**) ZIKV^Af^ strain in undifferentiated, D0- and D5-hNPCs were determined by standard plaque assay. Metabolic activity of undifferentiated, D0- and D5-hNPCs infected with the (**C**) ZIKV^As^ strain and (**D**) ZIKV^Af^ strain were determined using Wst1 Cell Proliferation Assay. The percentage of infected cell metabolic activity was normalized to the mock-infected cells. Experiments were performed three times and the results represent mean fold increase ± standard error of the mean. Comparison between two groups was performed with a Student’s t-test with a *P*-value significant when *p < 0.05, **p < 0.01, ***p < 0.001.

Fetal brain-derived primary hNPCs were used in the present study to mimick ZIKV infection in the context of the cellular differentiation process into neurons and astrocytes. Undifferentiated hNPCs as well as hNPCs in the process of differentiation starting were infected with ZIKV at MOI 0.5. For cells in the process of differentiation, infection was performed either at the time of growth factor withdrawal (further named D0 hNPCs) or 5 days after growth factor withdrawal (further named D5 hNPCs). Changes in terms of gene expression levels associated with neurogenesis were analyzed between D0 and D5 of cell differentiation (Supplementary Figure S1). In order to identify potential phenotypical differences, Asian (ZIKV^As^) and African (ZIKV^Af^) ZIKV strains were used. In hNPCs infected with the ZIKV^As^ strain, an increase of ZIKV titers was observed from 24 hpi to 96 hpi in undifferentiated and D0-hNPCs. An increase in the production of viral particles in ZIKV^As^ strain-infected D5-hNPCs was observed at 24 and 48 hpi (Figure 1A), whereas viral titers increased from 24 to 72 hpi in all states of hNPCs differentiation, infected with the ZIKV^Af^ strain (Figure 1B). Significant differences in viral production according to the cellular differentiation state were observed. Undifferentiated hNPCs were more permissive to ZIKV, as compared to D5-hNPCs from 24 hpi to 96 hpi (Figure 1A and B). We observed significant differences in viral production, in a cellular differentiation-dependent manner, with undifferentiated hNPCs producing the most important quantity of virus. Next, we determined cell viability by measuring metabolic activity in hNPCs-infected cells at different differentiation states (Figure 1C and 1D). ZIKV infection induces an increase of 25% of cellular proliferation in D5-hNPCs from 24 to 48 hpi, as compared to mock-infected cells. The same results were obtained with D0-hNPCs from 72 to 96 hpi with ZIKV^As^ strain only. In contrast, however, significant cell death or inhibition of proliferation was observed at 72 and 96 hpi, especially in infected undifferentiated cells, as compared to mock-infected cells. Comparison of metabolic activity induced by both strains revealed that ZIKV^Af^ (Figure 1D) infection was considerably more cytotoxic than that of the ZIKV^As^ strain (Figure 1C) in all stages of differentiation and in a time-dependent manner. Of note, in hNPCs infected with the African strain, significant diminution of metabolic activity was associated with high levels of viral production at 72 and 96 hpi, pointing to a strong positive correlation between cell viability and production of viral particles. Taken together, these results show that hNPC permissiveness to ZIKV infection differs according to cell differentiation state and to the nature of the viral strain.

### ZIKV infection induces premature differentiation in hNPCs

**Figure 2.**
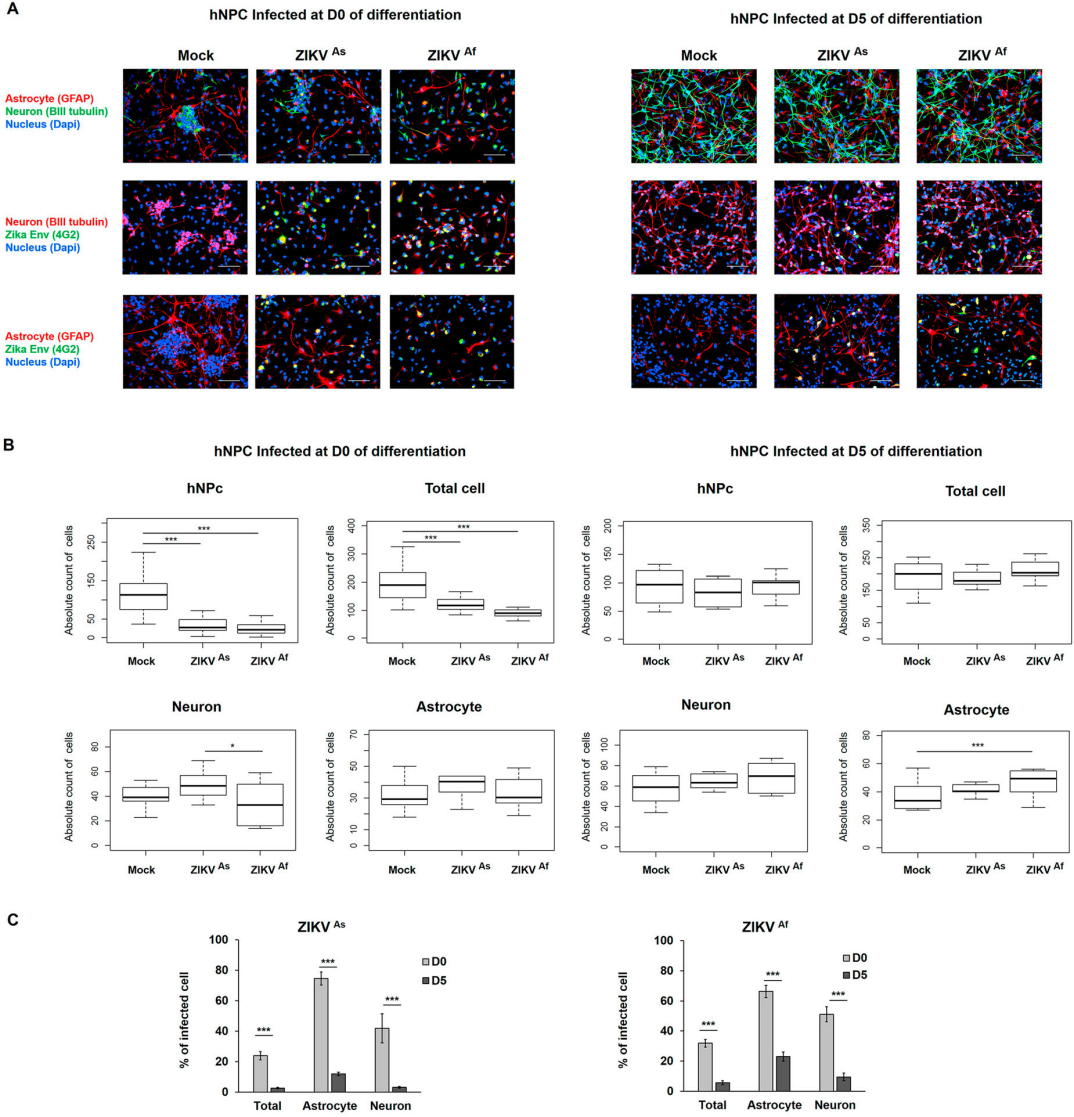
Distribution of cell populations in ZIKV-infected D0- and D5-hNPCs. Immunostaining of ZIKV infected hNPC in process of differentiation with antibodies directed against βIII-Tubulin (neuronal marker, red), GFAP (astrocyte marker, red) and 4G2 (Flavivirus marker, green) at 72 hpi, scale bar 100 µm. Nuclei were stained with DAPI (blue) (**A**). Distribution of absolute counts of neurons, astrocytes, hNPCs and total cells was determined within 10 independent squares of 0.2 mm^2^, using box plot (**B**). Comparison between two groups was performed with a Tuckey’s range test with a *P*-value significant when *p < 0.05, **p < 0.01, ***p < 0.001. The percentage of infected cells were manually determined based on virus markers (**C**). Comparison between two groups was performed with a Mann-Whitney test with a *P*-value significant when *p < 0.05, **p < 0.01, ***p < 0.001.

In order to explore the impact of ZIKV infection on the differentiation process in hNPCs, we carried out a cell population analysis in these cells following infection and subsequent differentiation. At D0 of differentiation, the high majority of cells are neural progenitors. However, few neurons and astrocytes are present, showing that few cells have already acquired a differentiated stage. At D5 of differentiation, we observed sensibly less hNPCs and astrocytes than at D0, yet more neurons, cellular extensions and therefore enhanced cellular interaction (Figure 2A). Enumeration of these cells did not show any significant difference in ZIKV-infected cultures, as compared to non-infected cultures, which indicates that the cytotoxic effect of ZIKV infection mainly affects undifferentiated hNPCs but not those at D5 of differentiation (Figure 2A and B). In fact, at the latter stage of differentiation no significant differences between Mock and ZIKV infected hNPC populations were observed, whereas the growth of undifferentiated, ZIKV-infected hNPCs significantly decreased, as compare to those that were mock-infected (Figure 2B). This difference in cytotoxic effects is specific for astrocytes and neurons that surround hNPCs which are more mature at D5, as compared to D0 of differentiation, and therefore could protect hNPCs through an emerging immune response. There was also an increase in the proportions of neurons and astrocytes in infected D5-hNPCs, as compared to uninfected cells, with a statistically significant result for astrocytes in D5-hNPCs infected with the ZIKV^Af^ strain (Figure 2B). Next, we evaluated the ZIKV-infected cell populations and confirmed that the entire D5 hNPC population was less permissive to ZIKV infection than D0-hNPCs. More specifically, we observed that astrocyte and neurons were more permissive to the infection at early stages of differentiation (Figure 2C).

### Innate immune response against ZIKV depends on hNPCs state of differentiation

**Figure 3.**
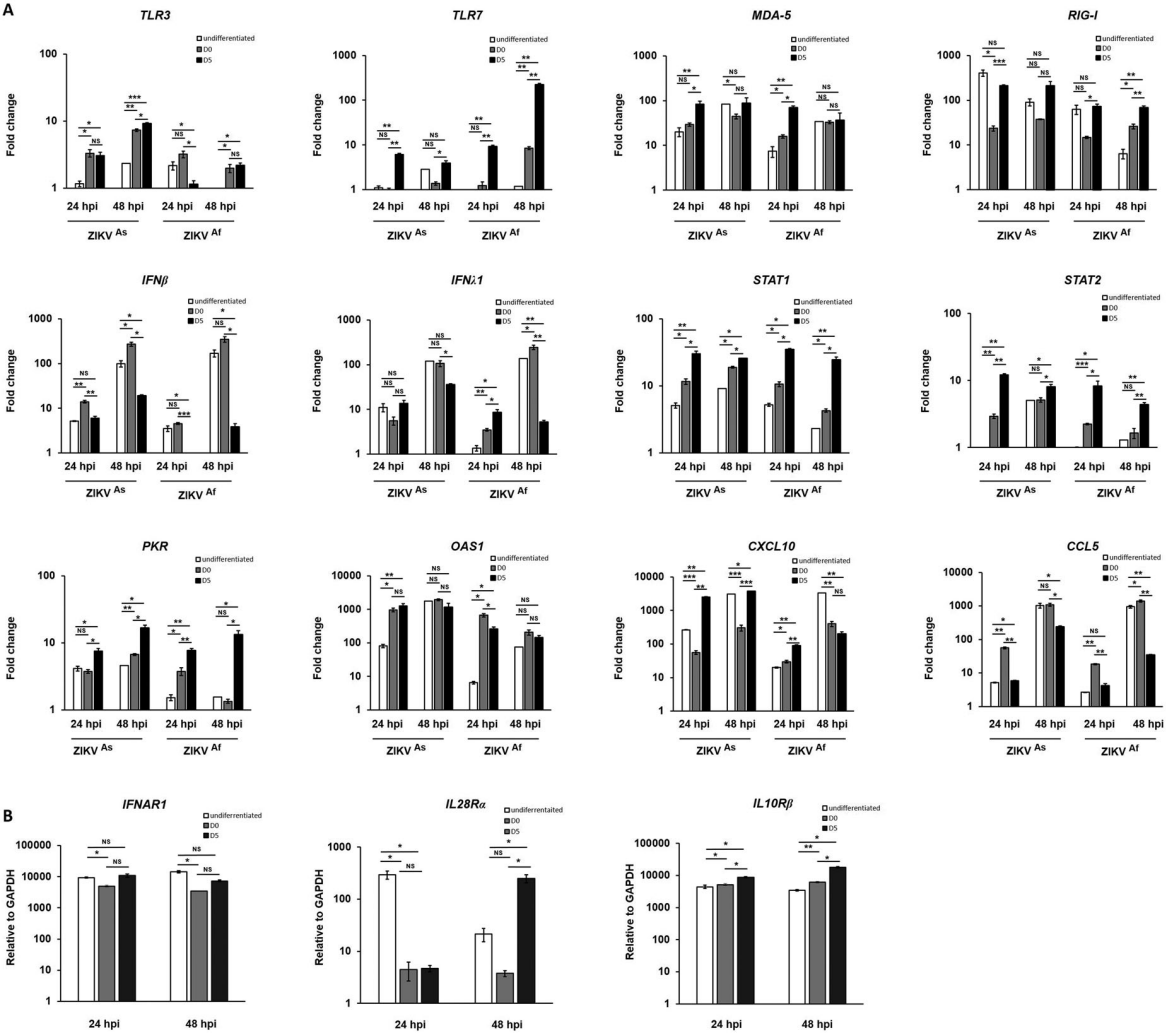
Modulation of innate immune gene expression by ZIKV in infected undifferentiated or D5-hNPCs. Total RNA from infected and uninfected hNPCs were analyzed for mRNA expression of PRRs, IFNs, ISGs, chemokines genes (**A**). Total RNA from uninfected hNPCs were analyzed for mRNA expression of IFNα/β receptor (IFNAR1) and IFNλ receptors (IL10Rβ and IL28Rα) (**B**). GAPDH was used as housekeeping gene for normalization. Experiments were performed three times and the results represent mean fold increase ± standard error of the mean. Comparison between two groups was performed with a Student’s t-test with a *P*-value significant when *p < 0.05, **p < 0.01, ***p < 0.001.

**Figure 4.**
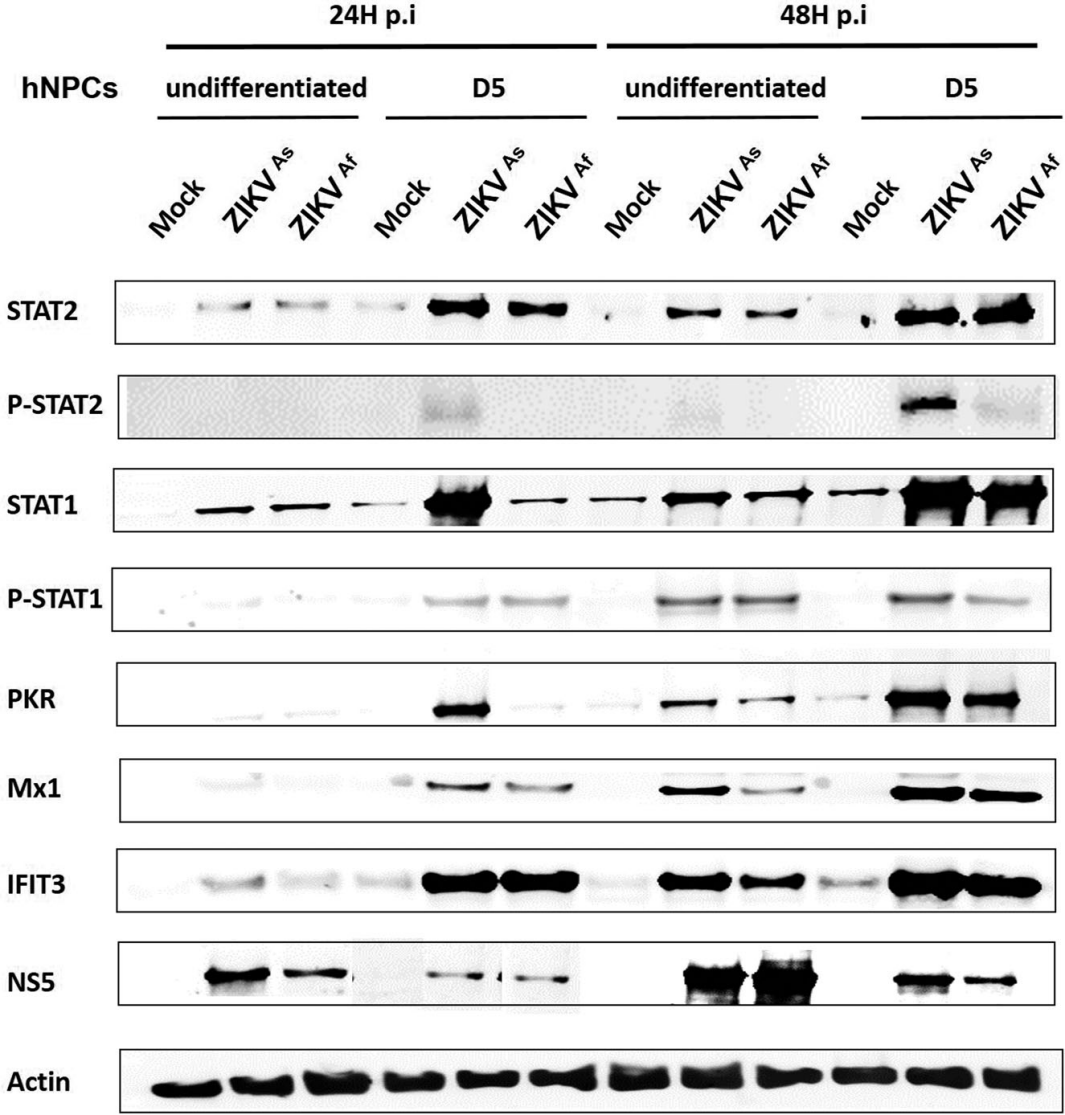
Innate immune response activation in undifferentiated or D5-ZIKV infected hNPCs. Expression of STAT2, pSTAT2, STAT1, pSTAT1, PKR, MX1, IFIT3 and NS5 was determined by Western blotting analysis in cell lysates of infected (24 and 48 hpi) and uninfected hNPCs. β-actin served as loading control. Results are representative of three independent experiments.

Based on the finding that the state of differentiation of hNPCs determines the degree of permissiveness to infection with ZIKV, we hypothesized that this might be due to differences in the innate immune response. Therefore, we compared the innate immune response induced by both ZIKV strains in undifferentiated, D0-hNPCs or D5-hNPCs (Figure 3). First, the expression of several TLR and RLR-associated genes involved in virus recognition was analyzed. The expression of TLRs including TLR3 and TLR7 genes was significantly higher in ZIKV-infected D5-hNPCs, as compared to undifferentiated cells (Figure 3A; and supplementary Figure S2). In addition, differences were observed depending on the infection with each of the viral strains. Indeed, ZIKV^As^ strain mainly induced the expression of TLR3, whereas that of TLR7 was mainly induced by ZIKV^Af^ strain. Then we analyzed the expression of MDA-5 and RIG-I (RLRs) genes, which recognize viral double strand RNA [32]. Significant differences were observed only at 24 hpi for MDA-5, suggesting that ZIKV infection with either strain induces MDA-5 gene expression earlier in D5-hNPCs, as compared to undifferentiated cells. In contrast, significant differences in RIG-I gene expression were observed only at 48 hpi, suggesting that ZIKV infection maintained RIG-I gene expression longer in D5-hNPCs than undifferentiated ones. Since the activation of these cellular receptors leads to the production of interferon [33], we assessed the expression of IFN type I (α,β) II (γ) and III (λ) genes. The main IFNs upregulated during infection of hNPCs with ZIKV were IFN-β and IFN-λ (Figure 3A), as compared to IFN-α and IFN-γ (supplementary Figure S2). In contrast to TLRs, the upregulation of IFN-β and IFN-λ expression was more pronounced in infected undifferentiated hNPCs, as compared to D5-hNPCs. Next, we investigated IFN signalling, which is associated with the activation of the Jak/Stat pathway. STAT2 gene expression was found to be strongly increased in infected D5-hNPCs, as compared to undifferentiated hNPCs (Figure 3). Similar findings were observed in ISG (Interferon Stimulated Gene) expression (Figure 3A), most notably with PKR, OAS1 at 24 hpi and Mx1 only at 48 hpi with ZIKV^Af^ strain (supplementary Figure S2). Although the expression of CCL5 and CXCL10, two chemokines associated with IFN signalling, was somewhat higher in D5-hNPCs at 24 hpi, as compared to undifferentiated and D0 cells, their expression profile was inversed at 48 hpi with undifferentiated hNPCs expressing the highest transcription levels for both genes. Because of the differences observed between IFN- and STAT gene expression we investigated the transcriptional levels of the IFN type I and type III receptors IFNAR1, IL28RA and IL10Rβ, respectively, in uninfected undifferentiated, D0- and D5-hNPCs (Figure 3B). The results show that IFN type III receptors were expressed at higher levels in D5-hNPCs than in undifferentiated and D0 hNPCs. Overall, the ZIKV^As^ strain induced higher levels of the genes involved in the innate immune response of hNPCs, as compared to the ZIKV^Af^ strain. Next, we evaluated whether the observed differences in STAT and ISG mRNA expression notably between infected undifferentiated and D5 hNPCs led to corresponding differences with respect to STAT phosphorylation and ISG protein levels (Figure 4 and supplementary Figure S4). Indeed, ZIKV infection resulted in an earlier and stronger induction of STAT phosphorylation, activation and production of antiviral proteins in D5-hNPCs, as compared to undifferentiated cells, which was associated with lower levels of the viral NS5 protein. Therefore, STAT gene expression levels depended on the differentiation state of the cells and became more intense in differentiated hNPCs. Taken together, our results demonstrate that D5-hNPCs mount a more robust innate immune response against ZIKV, as compared to infected-undifferentiated cells, which is associated with increased expression of IFN type III receptors.

### ZIKV infection dysregulates the Notch pathway during hNPC differentiation

**Figure 5.**
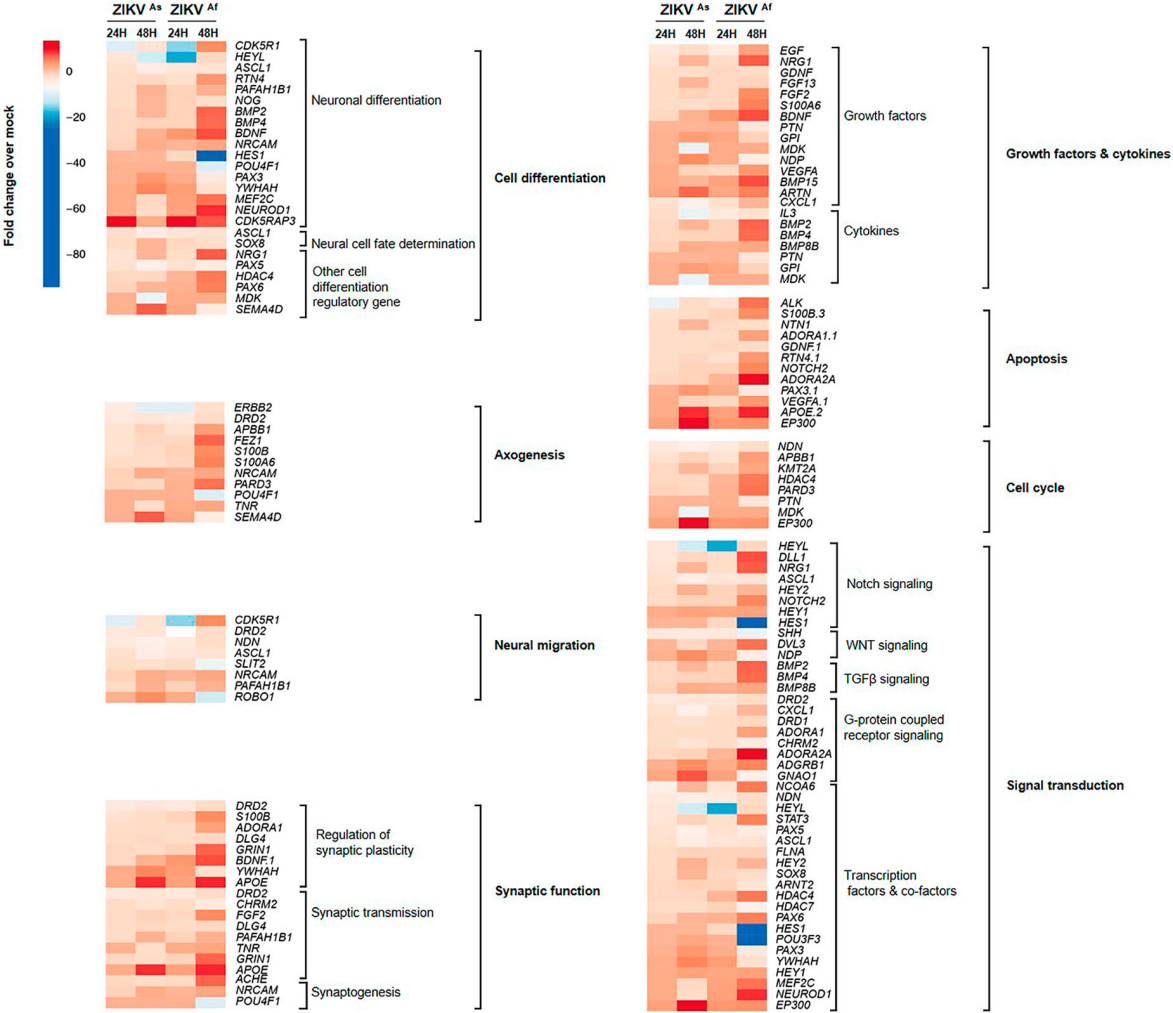
Neurogenesis RNA array analysis in ZIKV-infected hNPCs in the process of differentiation. Heatmap showing statistically significant up- (red) and downregulation (blue) of gene expression involved in neurogenesis pathways in ZIKV**^As^** and ZIKV^Af^ strain-infected hNPCs in process of differentiation, related to mock-infected cells at 24 and 48 hpi. Statistical analysis was performed using the RT2 profiler RT-PCR Array Data Analysis version 3.5.

**Figure 6.**
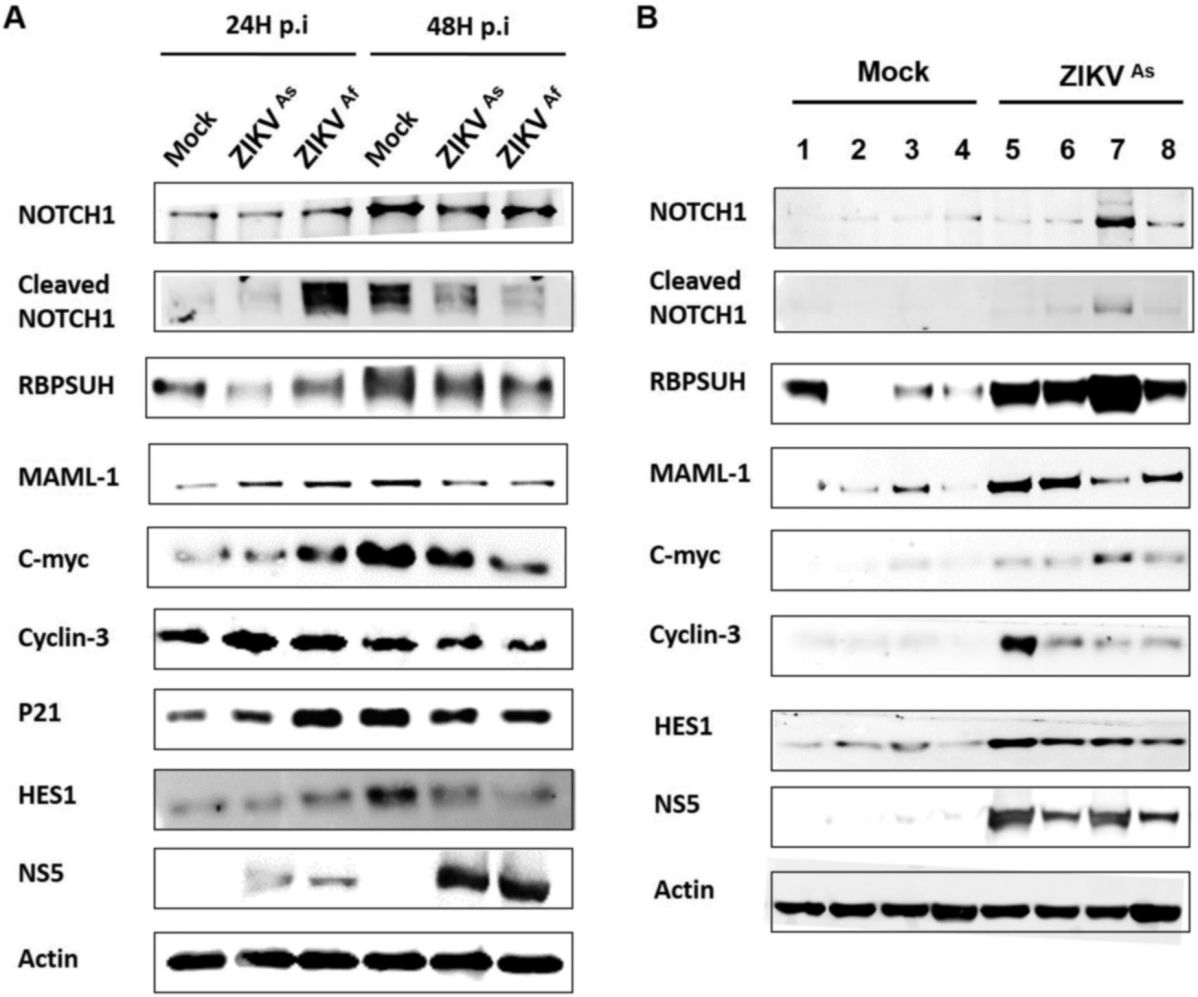
Western blot analysis of Notch pathway regulation in ZIKV infected D0-hNPCs and mouse brain. Cell lysates of mock or ZIKV-infected D0-hNPCs (A) and mock-(lines from 1 to 4) or ZIKV^As^-infected (lines from 5 to 8) mouse brain samples (B) were analyzed for Notch1, cleaved Notch1, RBPSUH, MAML-1, C-myc, Cyclin-D3, P21, Hes1, NS5 and β-actin as loading control. Results are representative of three independent experiments.

Recent studies have highlighted that gene expression changes in hNPCs exposed to ZIKV [20,34] are associated with the cell cycle, neural migration, differentiation and DNA replication and repair. In order to further understand the transcriptional changes occurring in ZIKV-infected hNPCs undergoing differentiation, we carried out a transcriptomic analysis targeting 84 genes that are crucial in neurogenesis, in particular those involved in cell cycling, neural migration, differentiation and DNA replication and repair. Transcripts from D0-hNPCs infected with ZIKV^As^ and ZIKV^Af^ strain at a MOI of 0.5 for 24 and 48 h were pooled from biological triplicates and compared to their matched mock-infected controls. ZIKV infection by both strains lead to a general change of expression of genes involved in neurogenesis (Figure 5), in particular the expression of genes involved in neural migration and differentiation. The expression of several genes involved in neurogenesis pathways, such as neuronal differentiation, regulation of synaptic plasticity, apoptosis, synaptic transmission and Notch signalling was induced to a greater extent by the ZIKV^Af^ strain, as compared to the ZIKV^As^ strain. Notch signalling regulates cell-fate determination during development and maintains adult tissue homeostasis and is involved in cell differentiation, proliferation and apoptosis, which represent the main cellular mechanisms affected by ZIKV infection. The modulation of several Notch pathway-associated genes expression, including HEYL, NOTCH2, HEY1, ERB2, ASCL1 and SHH genes was confirmed by quantitative PCR (supplementary Figure S3). Moreover, an accumulation of cleaved Notch, RBPSUH and MAML1, that are three gene products associated with the activation of the Notch pathway, as well as the target gene proteins c-myc, p21, cyclin-3 and Hes1, was observed in infected hNPCs at 24 hpi as compared to mock-infected cells (Figure 6A and supplementary Figure S5A). This overexpression was more significant in cells infected with the ZIKV^Af^ strain. However, the expression of these proteins was lower in ZIKV-infected cells at 48 hpi, as compared to mock-infected cells. To consolidate these results, we evaluated the expression of Notch signalling-associated proteins in mouse brain infected with the ZIKV^As^ strain of ZIKV (Figure 6B and supplementary Figure S5B). As compared to mock-infected mice, a significant upregulation of Notch pathway-related proteins was observed in the infected brains of E14.5 mouse embryos that were infected by intraplacental injection of ZIKV [29]. Taken together, these results suggest that ZIKV infection interferes with neuronal differentiation by dysregulating the canonical Notch signalling pathway, both in cultures of hNPCs and in mouse embryonic brains.

### Inhibition of the NOTCH pathway decreases ZIKV infection, rescues cell viability and modulates cell differentiation

**Figure 7.**
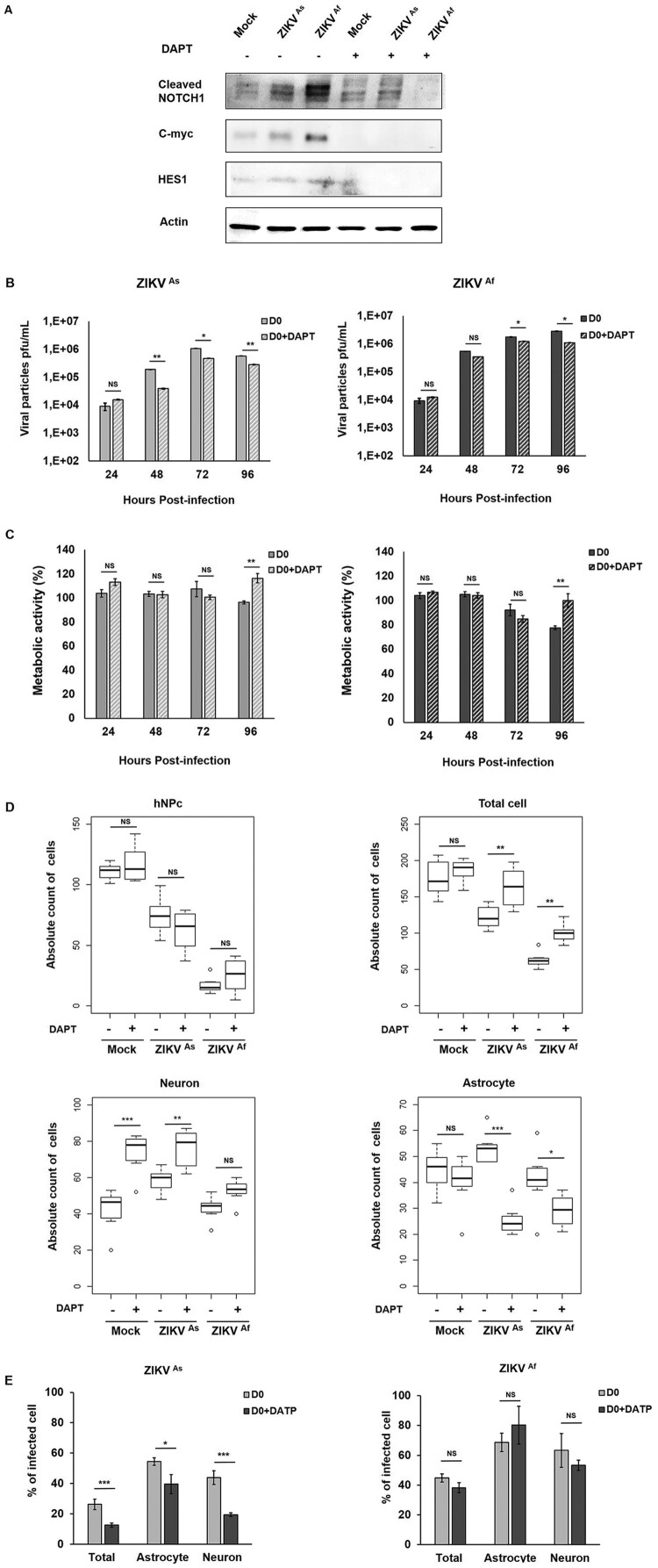
Notch inhibitor effect on ZIKV infectivity, cytotoxicity and cell differentiation in D0-hNPCs. Cell lysates of mock and ZIKV-infected D0-hNPCs treated with DAPT or untreated were analyzed for cleaved Notch1, C-myc and Hes1 (A). Distribution of absolute counts of neurons, astrocytes, hNPCs and total cells was determined within 10 independent squares of 0.2 mm^2^, using box plot (C). Comparison between DAPT treated and untreated cells was performed with a Tuckey’s range test with a *P*-value significant when *p < 0.05, **p < 0.01, ***p < 0.001. The percentage of infected cells were manually determined based on virus markers (**C**). Comparison between DAPT treated and untreated cells was performed with a Mann–Whitney test with a *P*-value significant when *p < 0.05, **p < 0.01, ***p < 0.001.

In order to evaluate whether Notch signalling or modulation of Notch signalling is a pre-requisite for ZIKV-induced pathogenesis, ZIKV infected or uninfected D0 hNPc were treated with a Notch inhibitor (DAPT). The inhibition of Notch signalling was evaluated by measuring the expression of Notch-associated proteins by Western blot analysis (Figure 7A and supplementary Figure S6). Inhibition of the Notch pathway led to a strong decrease in the expression of Cleaved Notch, C-myc and Hes1, at 24 hpi. Interestingly cells treated with DAPT and infected with the ZIKV^As^ of ZIKV produced less viral particles than untreated from 48 to 96 hpi (Figure 7B and C). Similar results were obtained with the ZIKV^Af^ strain, although but only between 72 and 96 hpi (Figure 7B). Moreover, DAPT treatment was found to significantly rescue the cell viability of hNPCs infected with either ZIKV strain at 96 hpi (Figure 7C). Numbers of ZIKV-infected D0-hNPCs cultured in the presence of DAPT were higher, as compared to untreated cells, thus confirming that inhibition of the Notch signalling pathway protected the cells from cytotoxicity induced with these ZIKV strains (Figure 7D). DAPT treatment did not have any effect on uninfected and ZIKV infected hNPcs whereas it strongly impacted the viability of differentiated cells, thereby suggesting that, ZIKV infection counteracts the effects of Notch inhibition by interfering with the differentiation of neurons and astrocytes. Finally, we confirmed that DAPT treatment inhibits infection by significantly decreasing the number of ZIKV^As^ infected cells but not of ZIKV^Af^ infected cells (Figure 7E).

## Discussion

The first concern of ZIKV infection is the emergence of congenital microcephaly and neurological disorders that are associated with defects in neurogenesis [8]. Although it has been previously shown that ZIKV exhibits neurotropism [19], the nature of the cellular pathways involved in neurogenesis and innate immune responses associated with ZIKV neuropathogenicity, has to be determined. Here, we show that hNPCs permissiveness to ZIKV infection is associated with the differentiation state of the infected cell, showing that the degree of susceptibility to ZIKV infection is inversely correlated with the differentiation state of the targeted cell. Moreover, the cytotoxic effects associated with viral infection were also found to be decreased during the neural differentiation process. Several studies have shown that ZIKV infection can have variable cytotoxic effects according to the viral strain and the cell type [35,36]. These results confirm and extend previous reports showing that ZIKV is able to directly infect human cortical neural progenitor cells and that that these cells are more susceptible to ZIKV infection than mature neurons and astrocytes [16,37] which is often associated with a diminution in the numbers of neural stem cells, resulting in a reduction of brain size [29]. This phenomenon could explain, at least in part, why ZIKV infection has more dramatic effects on brain development when the mother is infected during the first trimester of pregnancy [6,13,38–40]. Differences between both strains were also observed with the ZIKV^Af^ strain being more cytotoxic in undifferentiated and D0-hNPCs, as compared to ZIKV^As^ strain. These results sustain the hypothesis that the ZIKV^As^ strain is less virulent than the ZIKV^Af^ strain [36]. Together, these data also lead to the question as to why undifferentiated hNPCs are more susceptible to ZIKV infection than differentiated cells. The first possibility is that receptors involved in the efficient entry of ZIKV in cells are expressed on the membranes of neuronal progenitor cells and that their expression is lost or decreased during the differentiation process. This hypothesis is sustained by our results since we observed significant differences in viral production at the early time point (24 hpi) during differentiation, particularly with the ZIKV^As^ strain. Many studies have shown that AXL [13,22,41–43], but also DC-SIGN, Tyro3 and TIM-1 [22], promote the entry of the virus in different cell types. For example, Axl appears to be expressed on proliferating epithelial cells in the brain, but not in mature neurons [41]. These results notwithstanding, Axl seems not to be essential for ZIKV entry [44,45]. Therefore, further work is needed to identify additional receptors involved in ZIKV neurotropism and stem cell selectivity. Alternatively, it can be proposed that the differential capacity of hNPCs to induce an innate immune response to counteract ZIKV infection is dependent on the differentiation state of the infected cells. In order to test this hypothesis, we compared the capacity to induce an innate immune response between infected undifferentiated hNPCs and hNPCs infected at day 5 of differentiation. Gene expression analysis highlighted a globally stronger upregulation of innate immune response genes in infected D5-hNPCs than in undifferentiated hNPCs. More specifically, we observed that the TLR gene expression profile was upregulated to a greater degree in ZIKV-infected D5-hNPCs, as compared to undifferentiated cells. Moreover, we confirmed that the ZIKV^As^ strain preferentially induced TLR3 expression, whereas the ZIKV^Af^ strain mainly induced TLR7 expression in infected D5-hNPCs, as previously observed in ZIKV infected astrocytes [23]. These results could provide an explanation for the specificity of the ZIKV^As^ strain to induce brain disorders, since TLR3 activation has been associated with neural progenitor cell depletion in ZIKV^MR766^-infected cerebral organoids [20]. We also show that RIG-I and MDA-5 are mainly upregulated in D5-hNPCs, as compared to undifferentiated cells, whereas the analysis of IFN gene expression profile indicated that IFN-β and IFN-λ are the two main IFN genes that are induced during ZIKV infection of hNPCs. However, the induction of IFN gene expression does not seem to follow that of the TLR genes studied here, since IFN-β and IFN-λ transcripts appear to be upregulated to a greater extent in infected undifferentiated hNPCs than in D5-hNPCs. The magnitude of the expression of genes whose expression is associated with the JAK/STAT signalling pathway also revealed a strong difference between the state of cell differentiation with infected D5-hNPCs, showing a stronger upregulation of STATs and ISG genes than infected undifferentiated hNPCs. These observations could be explained by differences in IFN receptor expression by these cells. Our results furthermore show that the cellular differentiation process leads to an increase of type III IFNs receptor expression, suggesting that might allow for a stronger innate immune response induction. Similar results were obtained by investigating the activation of the STAT proteins and the production of ISGs proteins. It has been shown that ZIKV is able to antagonize type I IFN through the inhibition of STAT1 phosphorylation and STAT2 depletion, as demonstrated in dendritic cells and a kidney cell line [24,25,46]. Therefore, we hypothesize that the lack of IFN type III receptors associated with IFN signalling hijacked by ZIKV, contributes to a higher permissiveness of undifferentiated hNPCs to ZIKV infection. These results suggest that the cell differentiation process confers protection against ZIKV infection. Finally, both hypotheses can be correlated since the activity of Axl has been shown to suppress innate immune responses [42,47]. Cells have developed other strategies to clear viral infection such as the production of chemokines, which leads to the recruitment of lymphocytes. Paradoxically, an uncontrolled regulation of chemokines can lead to the development of neurodegenerative disorders [48]. A recent study has shown that acute ZIKV infection is associated with a peak of CXCL10 and CCL5 production, in infected patients [49]. In the present study, we show that CXCL10 and CCL5 expression is strongly upregulated, particularly in infected undifferentiated hNPCs, suggesting that ZIKV infection induces a strong inflammatory response, which could lead to an immunocytoxic effect, particularly in undifferentiated cells. Through a transcriptomic analysis, this study also revealed a global modulation of the expression of genes involved in neurogenesis in ZIKV-infected hNPCs. Several pathways affected following ZIKV infection have already been reported in the literature, such as those involving in apoptosis, cell cycle progression, cytokine production and cell differentiation [8,17,50]. The present work focuses on the Notch pathway, which regulates cell-fate determination during neural development, via the modulation of the proliferative, apoptotic, cell migratory and differentiation processes [51–54]. The Notch pathway acts as an ON/OFF switch resulting in an oscillation of the expression of genes associated with the signalling [53,54]. This property could explain why our results do not show an up or down regulation, but rather a dysregulation, in a time-dependent manner, of the Notch signalling pathway in infected hNPCs. Some studies have already highlighted that viral infection can modulate the Notch signalling pathway leading to neurodevelopmental disorders [54,55]. For example, Human Cytomegalovirus infection disrupts Hes1 protein expression whose depletion leads to NPCs cell fate disturbance, proliferation suppression and abnormal differentiation [54], similar to that observed in ZIKV-infected NPCs. Our results corroborate previous studies showing that ZIKV infection induces premature astrocyte differentiation in neural stem cells [56,57] since we observed an increased in quantity of astrocytes number in infected D5-hNPCs, as compared to uninfected cells. Therefore, our results suggest that ZIKV infection may initiate early activation of the Notch pathway resulting in premature differentiation of hNPCs. In order to clarify whether ZIKV infection neuropathogenesis is related to the dysregulation of the Notch signalling pathway, we evaluated the impact of Notch γ-secretase inhibitor DAPT on cell infectivity, cytotoxicity and differentiation. Interestingly, the inhibition of the Notch pathway lead to an inhibition of viral production of both strains, associated with decreased infected cells only with ZIKV^As^. Therefore, the activation of the Notch signalling pathway in infected cells seems to facilitate ZIKV production. Moreover, DAPT treatment lead to rescue cell viability and by consequences restrict the quantity of cells death. Finally, in mock infected cells, the inhibition of Notch lead to an increase differentiation in neurons, as it has been previously shown [58,59] but have no effects on the differentiation of astrocytes. In infected cells, the inhibition of Notch lead to an inhibition of cell differentiation in both neurons and astrocytes. Therefore, these results consolidate our hypothesis that ZIKV infection induced the activation of Notch pathway resulting in premature differentiation. It has indeed been shown that Notch signalling in response to activation of the TLR and JAK/STAT pathways promotes astrocyte fate from NPCs [53,60]. In view of our results showing that other TLRs, such as TLR4, TLR8 and TLR9, are upregulated during ZIKV infection, in particular with the ZIKV^As^ strain, additional studies are needed to determine the precise involvement of TLR activation in ZIKV pathogenicity. In conclusion, this study provides new molecular insight into ZIKV pathogenicity by highlighting the defect of undifferentiated cells to induce a strong innate immune response. Moreover, these data indicate that ZIKV infection potentially affects NPCs self-renewal and cell fate determination through the dysregulation of the Notch signalling pathway and activation of TLRs.

## Supplementary Material

Supplemental Material
