# Supplementary figures and images for: Zika virus differentially infects human neural progenitor cells according to their state of differentiation and dysregulates neurogenesis through the Notch pathway
Source: Emerg Microbes Infect. 2019 Jul 8;8(1):1003–16. doi: 10.1080/22221751.2019.1637283 (PMC6691766; doi:10.1080/22221751.2019.1637283)

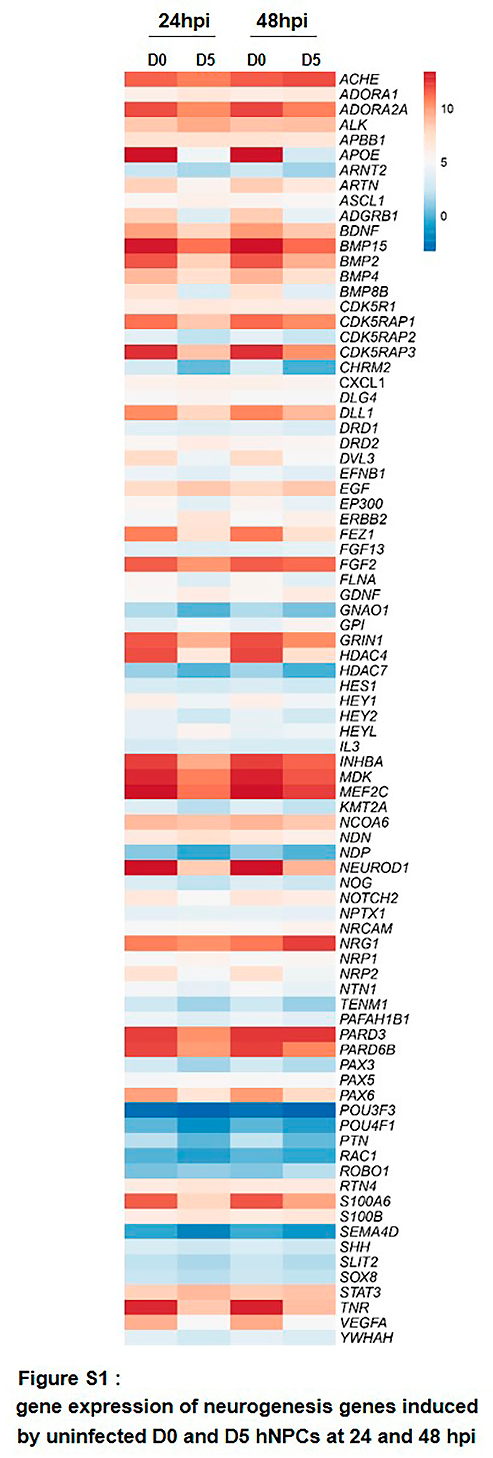

Supplement: Supplemental Material [file TEMI_A_1637283_SM5108.zip › Figure S1.png]

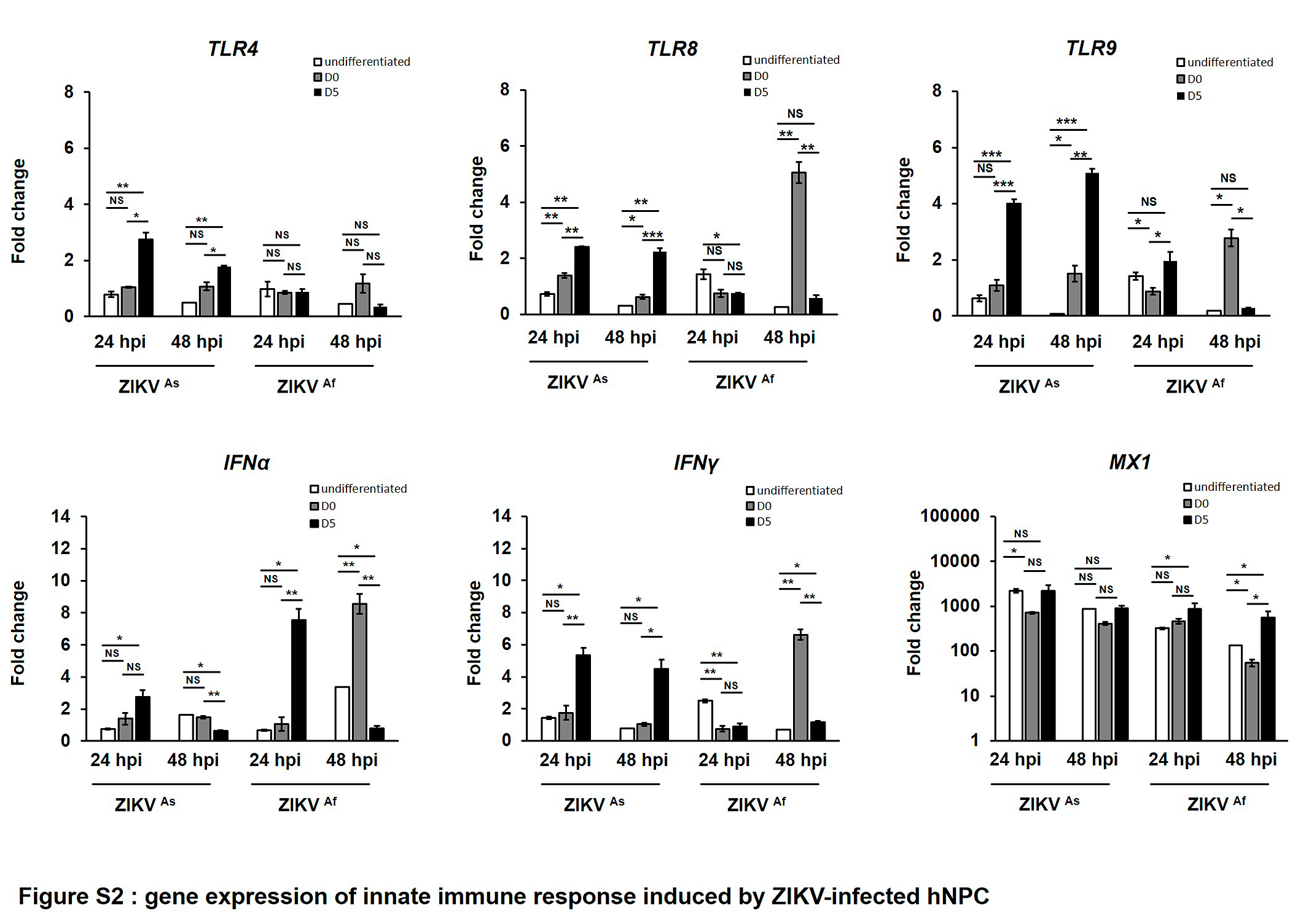

Supplement: Supplemental Material [file TEMI_A_1637283_SM5108.zip › Figure S2.png]

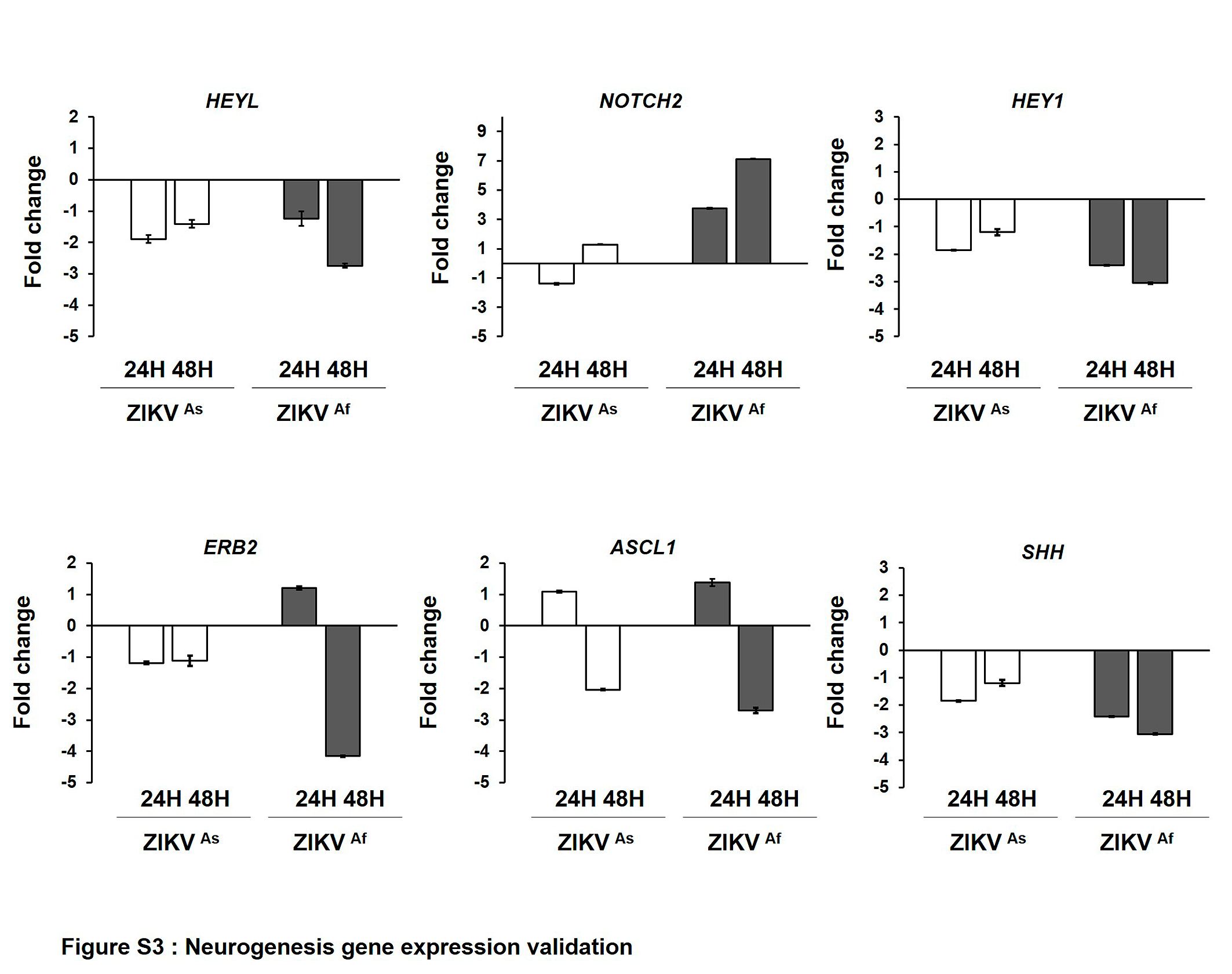

Supplement: Supplemental Material [file TEMI_A_1637283_SM5108.zip › Figure S3.png]

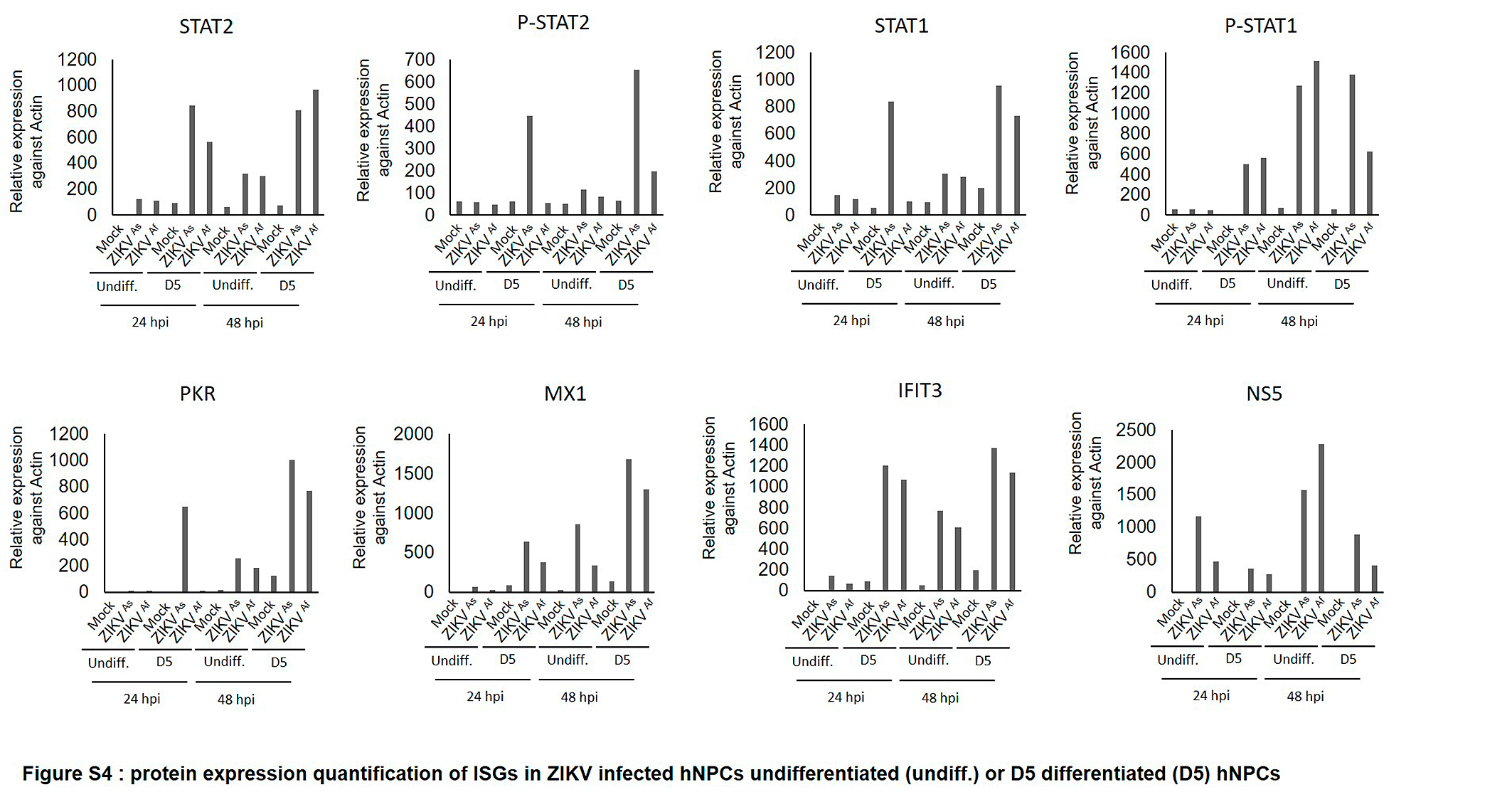

Supplement: Supplemental Material [file TEMI_A_1637283_SM5108.zip › Figure S4.png]

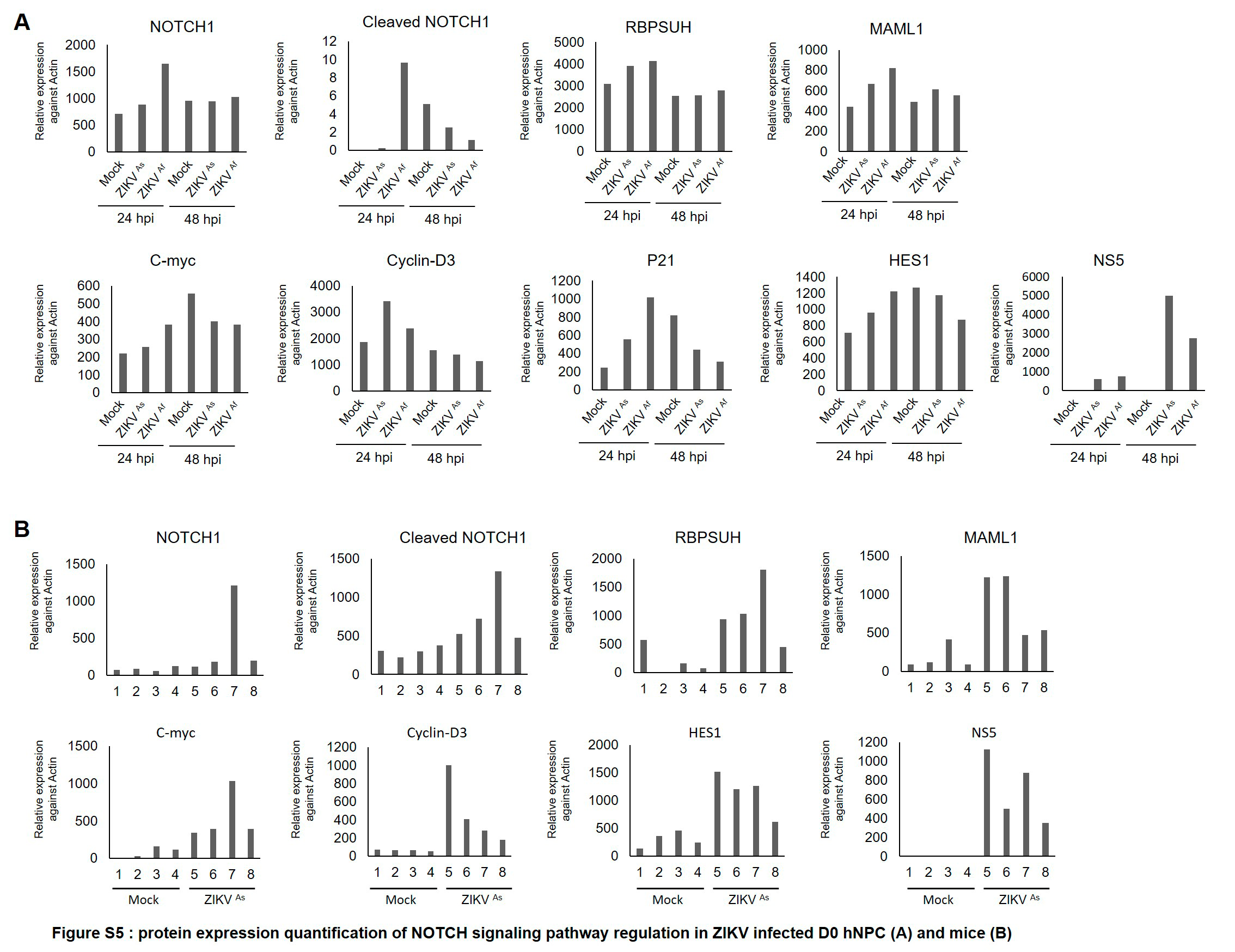

Supplement: Supplemental Material [file TEMI_A_1637283_SM5108.zip › Figure S5.png]

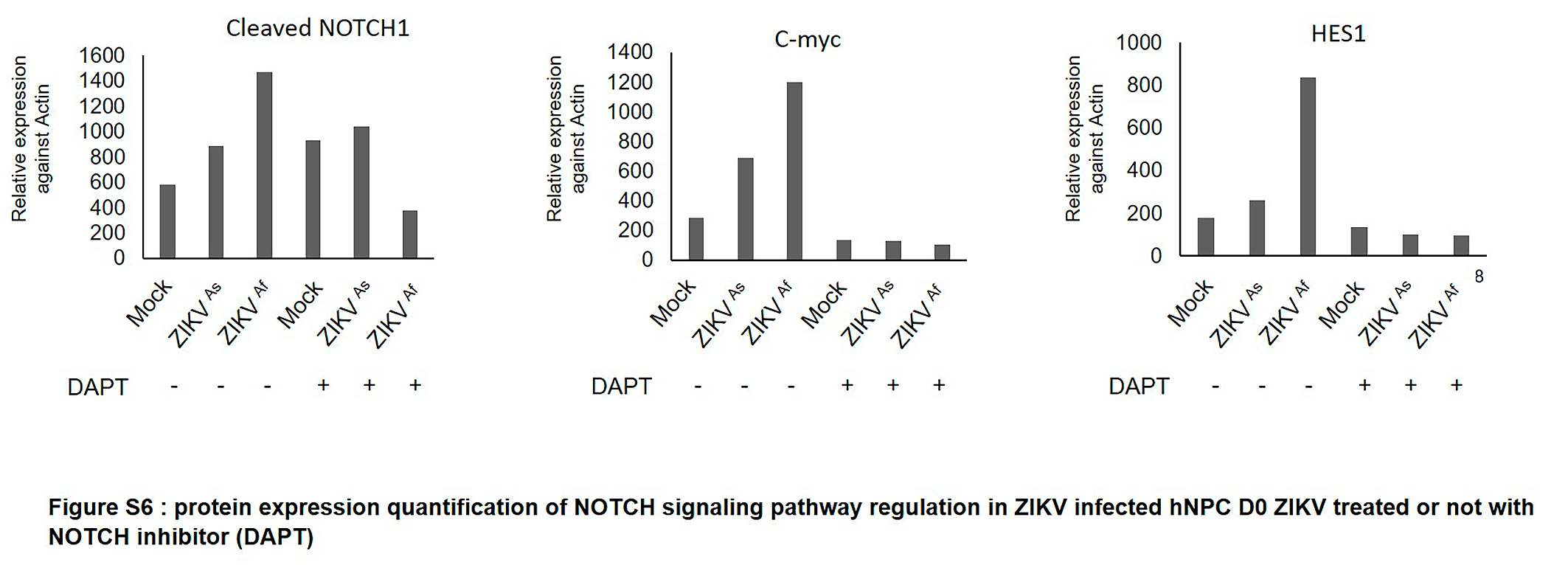

Supplement: Supplemental Material [file TEMI_A_1637283_SM5108.zip › Figure S6.png]
